# Correction: Azam et al. Removal of Chromium(III) and Cadmium(II) Heavy Metal Ions from Aqueous Solutions Using Treated Date Seeds: An Eco-Friendly Method. *Molecules* 2021, *26*, 3718

**DOI:** 10.3390/molecules31132240

**Published:** 2026-06-25

**Authors:** Mohammad Azam, Saikh Mohammad Wabaidur, Mohammad Rizwan Khan, Saud I. Al-Resayes, Mohammad Shahidul Islam

**Affiliations:** Department of Chemistry, College of Science, King Saud University, P.O. Box 2455, Riyadh 11451, Saudi Arabia; swabaidur@ksu.edu.sa (S.M.W.); mrkhan@ksu.edu.sa (M.R.K.); sresayes@ksu.edu.sa (S.I.A.-R.); mislam@ksu.edu.sa (M.S.I.)

## Text Correction

There was an error in the original publication [1].

The last sentence of Section 2. Materials and Methods, 2.2. Instrumentation was an error and should be deleted.

A correction has also been made to Section 3. Results and Discussion, 3.1.2. Fourier-Transform Infrared Spectroscopic Analysis (FTIR).

The sentence “From these findings, it can be postulated that -OH or NH C=O and C-O functions were principally responsible for binding Cr(III) and Cd(II) over the surface via electrostatic interactions and lone-pair donation [32].” should be corrected to “From these findings, it can be suggested that -OH or NH, C=O, and C-O functions were mainly responsible for binding with Cr(III) and Cd(II) on the surface via electrostatic interactions and lone-pair donation. A similar mechanism for metal adsorption has been reported in the literature [32].”

## Supplementary Materials Correction

The figure below for Cd(II) adsorption should be included in the Supplementary Materials and was mentioned in Section 3, 3.1. Characterization, 3.1.2. Fourier-Transform Infrared Spectroscopic Analysis (FTIR).

**Figure S1:**
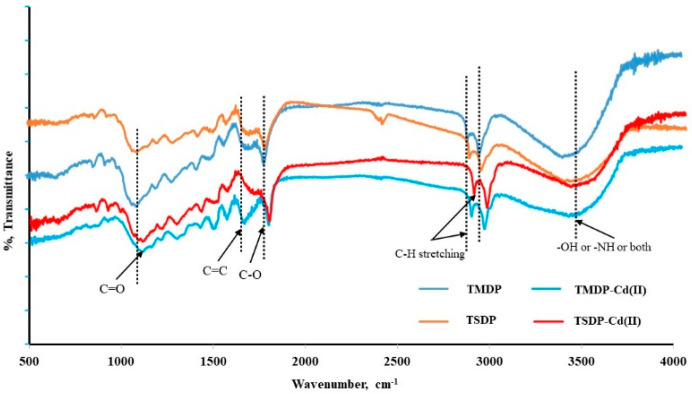
Cd(II) adsorption.

The authors state that the scientific conclusions are unaffected. This correction was approved by the Academic Editor and Editor-in-Chief. The original publication has also been updated.
