# Supplementary material for: Removal of Chromium(III) and Cadmium(II) Heavy Metal Ions from Aqueous Solutions Using Treated Date Seeds: An Eco-Friendly Method
Source: Molecules. 2021 Jun 18;26(12):3718. doi: 10.3390/molecules26123718 (PMC8235514; doi:10.3390/molecules26123718)
Supplement: Supplementary file 1 [file molecules-26-03718-s001.zip › molecules-1240360-supplementary.pdf]

molecules

Removal of Chromium(III) and Cadmium(II) Heavy Metal Ions from Aqueous Solutions Using Treated Date Seeds: An Eco-Friendly Method

Mohammad Azam *, Saikh Mohammad Wabaidur, Mohammad Rizwan Khan, Saud I. Al-Resayes and Mohammad Shahidul Islam

Department of Chemistry, College of Science, King Saud University, P.O. Box 2455, Riyadh 11451, Saudi Arabia; swabaidur@ksu.edu.sa (S.M.W.); mrkhan@ksu.edu.sa (M.R.K.); sresayes@ksu.edu.sa (S.I.A.-R.); mislam@ksu.edu.sa (M.S.I.)

* Correspondence: mhashim@ksu.edu.sa

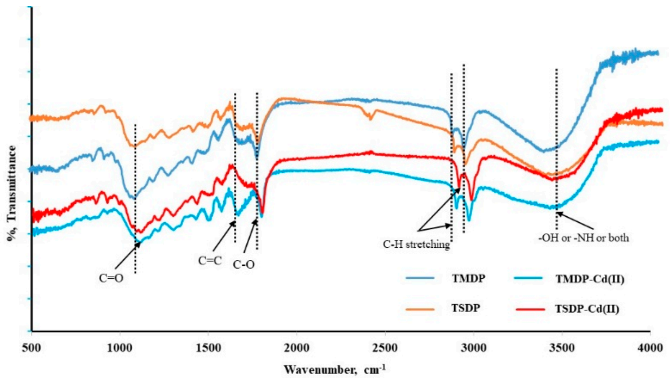

Figure S1. Cd(II) adsorption.

 https://doi.org/10.3390/molecules26123718
